# The increasing burden of testicular seminomas and non-seminomas in adolescents and young adults (AYAs): incidence, treatment, disease-specific survival and mortality trends in the Netherlands between 1989 and 2019

**DOI:** 10.1016/j.esmoop.2023.102231

**Published:** 2024-01-19

**Authors:** D.J. van der Meer, H.E. Karim-Kos, H.W. Elzevier, M. Dinkelman-Smit, J.M. Kerst, V. Atema, V. Lehmann, O. Husson, W.T.A. van der Graaf

**Affiliations:** 1 Department of Medical Oncology, Netherlands Cancer Institute—Antoni van Leeuwenhoek, Amsterdam; 2 Department of Psychosocial Research and Epidemiology, Netherlands Cancer Institute, Amsterdam; 3 Department of Medical Oncology, Erasmus MC Cancer Institute, Erasmus University Medical Center, Rotterdam; 4 Princess Máxima Center for Pediatric Oncology, Utrecht; 5 Department of Research and Development, Netherlands Comprehensive Cancer Organization (IKNL), Utrecht; 6 Department of Urology and Medical Decision Making, Leiden University Medical Centre, Leiden; 7 Department of Urology, Erasmus MC Cancer Institute, Erasmus University Medical Center, Rotterdam; 8 Department of Medical Psychology, Cancer Center Amsterdam, Amsterdam University Medical Center, Amsterdam; 9 Cancer Center Amsterdam (CCA), Amsterdam; 10 Department of Surgical Oncology, Erasmus MC Cancer Institute, Erasmus University Medical Center, Rotterdam, The Netherlands

**Keywords:** oncology, adolescent and young adult, incidence, mortality, survival, testicular cancer

## Abstract

**Background:**

Testicular cancer incidence among adolescents and young adults (AYAs, aged 18-39 years at diagnosis) is increasing worldwide and most patients will survive the initial disease. Still, detailed epidemiological information about testicular cancer among AYAs is scarce. This study aimed to provide a detailed overview of testicular cancer trends in incidence, treatment, long-term relative survival and mortality by histological subtype among AYAs diagnosed in the Netherlands between 1989 and 2019.

**Materials and methods:**

Data of all malignant testicular cancers (ICD-code C62) were extracted from the Netherlands Cancer Registry. Mortality data were retrieved from Statistics Netherlands. European age-standardized incidence and mortality rates with average annual percentage change statistics and relative survival estimates up to 20 years of follow-up were calculated.

**Results:**

A total of 12 528 testicular cancers were diagnosed between 1989 and 2019. Comparing 1989-1999 to 2010-2019, the incidence increased from 4.4 to 11.4 for seminomas and from 5.7 to 11.1 per 100 000 person-years for non-seminomas. Rising trends were most prominent for localized disease. Radiotherapy use in localized testicular seminomas declined from 78% in 1989-1993 to 5% in 2015-2019. Meanwhile, there was a slight increase in chemotherapy use. Most AYAs with localized seminomas and non-seminomas received active surveillance only (>80%). Overall, relative survival estimates remained well above 90% even at 20 years of follow-up for both seminomas and non-seminomas. Mortality rates declined from 0.5 to 0.4 per 100 000 person-years between 1989-1999 and 2010-2019.

**Conclusions:**

The incidence of seminoma and non-seminoma testicular cancers significantly increased in AYAs in the Netherlands between 1989 and 2019. There was a shift towards less-aggressive treatment regimens without negative survival effects. Relative survival estimates remained well above 90% at 20 years of follow-up in most cases. Testicular cancer mortality was already low, but has improved further over time, which makes survivorship care an important issue for these young adults.

## Highlights

• AYAs were diagnosed in near equal measure with seminomas (47.3%) and non-seminomas (52.4%).
• The incidence more than doubled for seminomas and almost doubled for non-seminomas between 1989-1999 and 2010-2019.
• Radiotherapy use in localized testicular seminomas declined from 78% in 1989-1993 to 5% in 2015-2019.
• Relative survival estimates remained well above 90% even at 20 years of follow-up for both seminomas and non-seminomas.
• Testicular cancer mortality slightly declined from 0.5 to 0.4 per 100 000 person-years between 1989-1999 and 2010-2019.

## Introduction

The incidence of cancer among adolescents and young adults (AYAs, aged 18-39 years at cancer diagnosis) in industrialized countries is increasing, survival is improving and overall mortality is declining, leading to a growing population of AYA cancer survivors.^1^, ^2^, ^3^, ^4^, ^5^ A major contributor to these trends is the prominent increase in testicular cancer that is observed among male AYAs at many places globally.^1^^,^^4^^,^^6^ Testicular cancer is the most common malignancy among young males of reproductive age, with the peak incidence occurring in individuals aged in their late 20s and 30s.^2^^,^^7^, ^8^, ^9^, ^10^ Testicular cancers predominantly consist of germ-cell tumours, which are categorized into seminoma and non-seminoma histological subtypes^7^^,^^8^^,^^10^ and have among the highest cure rates of cancer in AYAs, related to the hallmark success of cisplatin-based chemotherapy.^11^^,^^12^ The 5-year relative survival of testicular cancer overall exceeds 95%, meaning that almost all men survive the initial years following diagnosis.^1^^,^^2^^,^^13^ This excellent survival is independent of treatment strategy and to some extent also relates to most patients being diagnosed with early-stage disease that can be cured with orchiectomy alone when confined to the testis.^8^^,^^14^^,^^15^

Prenatal and postnatal exposure to both environmental endocrine disruptors (e.g. pesticides, solvents, personal care products)^16^, ^17^, ^18^, ^19^, ^20^, ^21^, ^22^, ^23^, ^24^ and genetic factors (e.g. BAK1, DMRT1, TERT-CLPTM1L, KITLG, androgen receptor gene and PDE11A polymorphisms)^21^^,^^25^ likely play an important role in testicular cancer carcinogenesis, but establishing clear associations has been challenging due to data scarcity and inconsistent evidence.^26^, ^27^, ^28^ While still debated, research suggests that exposure to environmental endocrine disruptors leads to testicular dysgenesis syndrome, which encompasses various clinical conditions, including hypospadias, cryptorchidism, infertility, low testosterone levels and testicular cancer.^16^, ^17^, ^18^, ^19^, ^20^, ^21^, ^22^, ^23^, ^24^ Environmental endocrine disruptors are by-products of industrialization and urbanization and would explain the distinct rise in testicular cancer in industrialized counties.^21^^,^^29^, ^30^, ^31^ Familial occurrence among first-degree relatives, contralateral testicular cancer^24^^,^^32^ and birth-related factors (e.g. low birth weight, low gestational age and low or high maternal age) are also commonly cited.^24^ Increased germ-cell testicular cancer risk with increased duration of employment among agricultural (e.g. animal husbandry workers), electrical and electronics and salesmen (e.g. retail, supermarket, non-specialty stores) workers was also observed in a recent paper from the TESTIS study group in France, reaffirming that exposure to occupational-related agents and chemicals are likely involved in disease development.^33^ Despite growing research on testicular cancer aetiology, a comprehensive understanding of the underlying causes and molecular mechanisms (e.g. exposure time to endocrine disruptors and genetic background) is still lacking,^21^^,^^32^^,^^34^ hampering large-scale preventative measures to stabilize the increasing incidence, such as legislation to decrease endocrine-disrupting chemicals in the environment or screening for individuals at risk for developing testicular cancer.

Adding to a lack of clear insight, detailed epidemiological literature about testicular cancer by age at diagnosis, tumour stage, treatment regimens and histological subtype in AYAs is limited and often restricted to short-term outcomes (e.g. 5-year survival). More epidemiological information on incidence and early and late survival rates of specific histological subtypes will not only meet the information needs of patients,^35^ but can also serve as relevant information during later life when specific AYA survivorship topics and late effects of treatment need to be encountered.

This study provides a detailed overview of subtype-specific trends in incidence, treatment, long-term relative survival (5-20 years) and mortality of AYAs (aged 18-39 years) diagnosed with testicular cancer in the Netherlands between 1989 and 2019.

## Materials and methods

### Data sources

Cancer incidence, treatment and survival data were obtained from the nationwide population-based Netherlands Cancer Registry (NCR), hosted by the Netherlands Comprehensive Cancer Organisation (IKNL). The NCR records data of newly diagnosed malignancies in the Netherlands with nationwide coverage since 1989 and based on notification by the National Network and Registry of Histopathology and Cytopathology (PALGA) and supplemented by information from the Dutch hospital database and various haematology laboratories. The NCR annually links their data to the nationwide Personal Records Database (BRP), which contains vital status information (dead, alive or emigrated) of all residents in the Netherlands. Linkage for this study occurred on 1 February 2021. Treatment information within the NCR is directly obtained from the medical records by trained registrars, ensuring good accuracy. The NCR categorizes neoplasms in accordance with the International Classification of Diseases for Oncology (ICD-O) based on its first edition (until 1993), second edition (1993-2000) and third edition (since 2001).^36^ Tumour stage is coded based on the Union for International Cancer Control (UICC) TNM (tumour–node–metastasis) classification of malignant tumours in its fourth edition (1989-1998), fifth edition (1999-2002), sixth edition (2003-2009), seventh edition (2010-2016), and since 2017 all tumours are coded based on the eighth edition.^37^ Cancer-specific mortality data were retrieved from the cause-of-death statistics of Statistics Netherlands (http://statline.cbs.nl).

### Data selection

From the above sources, data were extracted of all testicular cancers (ICD-code C62) with behaviour /3 that were diagnosed during adolescence and young adulthood (i.e., age 18-39 years) in the Netherlands between 1989 and 2019. Malignancies in this study were categorized according to the histology-based AYA classification scheme^38^^,^^39^ and further classified as localized (T1-4, N0/Nx, M0/Mx or T0/Tx, N0, M0), regional lymph nodes (any T, N+, M0/Mx), distant metastases (any T, any N, M1) and stage unknown. Clinical-stage data were used to supplement missing/unknown pathological-stage data. AYAs with missing/unknown stage information (*n* = 79, 0.6%) were excluded from the stage-specific analyses. Data of primary cancer surgery within the NCR are standard categorized into organ (i.e. orchiectomy, total resection and surgery not specified), local (i.e. excision and resection of tumour) and other types of surgery (i.e. additional resection). The ‘orchiectomy’ groups within this study consisted of organ surgery only, which may still contain—although unlikely for this tumour type—other surgery types due to older codes not specifying orchiectomy. Local and other types of surgery were included within the various ‘other’ groups. A ‘no treatment’ group (*n* = 15, 0.1%) was added for the few cases that did not receive any treatment. For international comparison of incidences, the international rules concerning multiple cancers were applied to the data.^36^ Mortality data of testicular cancers (ICD-9 code 186 between 1989-1995 and ICD-10 code C62 thereafter) were available for individuals aged 15-39 years from 1989 to 2019.

### Statistical analyses

Treatment proportions were calculated based on treatments received by patients at any time, irrespective of duration or completion.

Age-standardized incidence and mortality rates per 100 000 person-years with weights based on the 2013 revised European Standard Population were calculated.^40^ The mid-year population size was used as person-time denominator and was calculated from annual data of the Dutch general population size, obtained from Statistics Netherlands, by averaging consecutive years (http://statline.cbs.nl). Trend changes in incidence and mortality over the entire 30-year study period were evaluated by calculating the average annual percentage change (AAPC) and corresponding 95% confidence intervals (CIs) with the Joinpoint Regression Program (version 4.9.0.0) developed by the Surveillance, Epidemiology, and End Results (SEER) program. Calculations within Joinpoint were done using the grid search method, allowing three points between adjacent observed x-values, and the uncorrelated error model parameter setting.^41^ The allowed number of joinpoints per model varied between 0 and 5. Final model selection was based on the recommended Bayesian information criteria 3. The remaining program parameters were kept at their default setting.

The relative survival was used as an estimator for disease-specific survival and is the ratio between the observed survival in patients and the survival that is expected in a general population comparable in terms of sex and age in each calendar year. Annual expected survival probability data of the Dutch general population were retrieved from Statistics Netherlands (http://statline.cbs.nl). End of follow-up was defined as the year of death, emigration or 2019, whichever came first. Computation of relative survival was done with the -strs- command in Stata/SE 17.0 (StataCorp LP, College Station, TX) and using the Ederer II methodology.^42^ The traditional cohort-approach was used to calculate 5- and 10-year relative survival for all diagnostic periods, as well as up to 20-year relative survival for 1989-1999 and 2000-2009. For the latest diagnostic period (2010-2019), long-term 15- and 20-year relative survival outcomes were supplemented with the period-approach.^43^ Changes in relative survival by diagnostic period were evaluated by examining overlap of the 95% CIs. Two-sided *P* values <0.05 were considered statistically significant. Analyses were stratified by histological subtype and by period of diagnosis (1989-1999, 2000-2009, 2010-2019), age at diagnosis (18-24, 25-29, 30-34, 35-39 years) and tumour stage. The study was approved by the Privacy Review Board of the NCR. Data used in this study can be requested from the NCR (request number: K21.058).

## Results

### Population, tumour and treatment characteristics

Between 1989 and 2019, a total of 12 528 AYA testicular cancers were diagnosed in the Netherlands. Nearly all were germ-cell cancers (99.7%), with an almost equal distribution between seminomas (47.3%) and non-seminomas (52.4%). Non-germ-cell testicular cancers were extremely rare (0.3% of cases). Median age at diagnosis was 32 years [interquartile range (IQR): 28-36 years] for seminomas and 27 years (IQR: 23-32 years) for non-seminomas. Most seminomas (83.7%) and non-seminomas (62.9%) were localized. At diagnosis, seminomas with distant metastases were found in 2.4% of cases, whereas for non-seminomas this was 13.7% (Table 1).

**Table 1:** Population, tumour and treatment characteristics of male adolescents and young adults (AYAs) diagnosed with testicular cancer at ages 18-39 years in the Netherlands between 1989 and 2019

Characteristics^a^	All testicular cancers	Seminomas					Non-seminomas				
Period of diagnosis	Total	Total	1989-1999	2000-2009	2010-2019	*P* value^b^	Total	1989-1999	2000-2009	2010-2019	*P* value^b^
	*n* (%)	*n* (%)	*n* (%)	*n* (%)	*n* (%)		*n* (%)	*n* (%)	*n* (%)	*n* (%)	
Total	12 528 (100.0)	5930 (100.0)	1474 (100.0)	1915 (100.0)	2541 (100.0)	NA	6560 (100.0)	1723 (100.0)	2238 (100.0)	2599 (100.0)	NA
Median age (IQR), years	30.0 (25.0-34.0)	32.0 (28.0-36.0)	32.0 (28.0-35.0)	32.0 (29.0-36.0)	32.0 (28.0-36.0)	0.073	27.0 (23.0-32.0)	27.0 (23.0-32.0)	27.0 (23.0-32.0)	27.0 (24.0-32.0)	0.177
Age group (years)											
18-24	2576 (20.6)	490 (8.3)	117 (7.9)	158 (8.3)	215 (8.5)	0.195	2082 (31.7)	555 (32.2)	733 (32.8)	794 (30.6)	0.899
25-29	3401 (27.1)	1342 (22.6)	366 (24.8)	407 (21.3)	569 (22.4)		2046 (31.2)	524 (30.4)	697 (31.1)	825 (31.7)	
30-34	3633 (29.0)	2137 (36.0)	536 (36.4)	703 (36.7)	898 (35.3)		1487 (22.7)	412 (23.9)	476 (21.3)	599 (23.1)	
35-39	2918 (23.3)	1961 (33.1)	455 (30.9)	647 (33.8)	859 (33.8)		945 (14.4)	232 (13.5)	332 (14.8)	381 (14.7)	
Tumour stage (TNM)^c^											
Localized	9091 (72.8)	4963 (83.7)	1195 (81.2)	1594 (83.3)	2174 (85.6)	<0.001	4125 (62.9)	972 (56.5)	1412 (63.1)	1741 (67.0)	<0.001
Regional lymph nodes	2087 (16.7)	773 (13.0)	208 (14.1)	259 (13.5)	306 (12.0)		1314 (20.1)	381 (22.2)	432 (19.3)	501 (19.3)	
Distant metastases	1271 (10.2)	175 (3.0)	58 (3.9)	57 (3.0)	60 (2.4)		1096 (16.7)	353 (20.5)	388 (17.3)	355 (13.7)	
Stage unknown	36 (0.3)	16 (0.3)	11 (0.8)	4 (0.2)	1 (0.0)		20 (0.3)	14 (0.8)	5 (0.2)	1 (0.0)	
Missing	43	3	2	1	0		5	3	1	1	
Treatment^d^											
Orchiectomy only (active surveillance)	5756 (45.9)	2169 (36.6)	189 (12.8)	339 (17.7)	1641 (64.6)	<0.001	3553 (54.2)	847 (49.2)	1216 (54.3)	1490 (57.3)	<0.001
Orchiectomy + CT (± other)^e^	3512 (28.0)	906 (15.3)	174 (11.8)	235 (12.3)	497 (19.6)		2606 (39.7)	757 (43.9)	878 (39.2)	971 (37.4)	
Orchiectomy + RT (± other)^f^	2665 (21.3)	2639 (44.5)	1023 (69.4)	1280 (66.8)	336 (13.2)		26 (0.4)	11 (0.6)	12 (0.5)	3 (0.1)	
Orchiectomy + RPLND/metastasectomy (± other)^g^	131 (1.0)	22 (0.4)	3 (0.2)	8 (0.4)	11 (0.4)		108 (1.7)	38 (2.2)	37 (1.7)	33 (1.3)	
Orchiectomy + other (± other)^h^	153 (1.2)	91 (1.5)	56 (3.8)	21 (1.1)	14 (0.6)		62 (1.0)	26 (1.5)	26 (1.2)	10 (0.4)	
CT only	32 (0.3)	11 (0.2)	6 (0.4)	2 (0.1)	3 (0.1)		19 (0.3)	6 (0.4)	7 (0.3)	6 (0.2)	
CT + orchiectomy (± other)^i^	181 (1.4)	49 (0.8)	12 (0.8)	17 (0.9)	20 (0.8)		132 (2.0)	28 (1.6)	43 (1.9)	61 (2.4)	
CT + other (± other)^j^	30 (0.2)	9 (0.2)	0 (0.0)	3 (0.2)	6 (0.2)		21 (0.3)	2 (0.1)	6 (0.3)	13 (0.5)	
Other	53 (0.4)	25 (0.4)	8 (0.5)	9 (0.5)	8 (0.3)		27 (0.4)	6 (0.4)	11 (0.5)	10 (0.4)	
No treatment	15 (0.1)	9 (0.2)	3 (0.2)	1 (0.1)	5 (0.2)		6 (0.1)	2 (0.1)	2 (0.1)	2 (0.1)	
Histological subtype											
Germ-cell tumours^k^	12 490 (99.7)	NA	NA	NA	NA	NA	NA	NA	NA	NA	NA
Non-germ-cell tumours	38 (0.3)	NA	NA	NA	NA	NA	NA	NA	NA	NA	NA
Carcinoma	7 (0.1)	NA	NA	NA	NA	NA	NA	NA	NA	NA	NA
Sex cord	31 (0.2)	NA	NA	NA	NA	NA	NA	NA	NA	NA	NA

CT, chemotherapy; ICD-O, International Classification of Diseases for Oncology; IQR, interquartile range; NA, not applicable; RPLND, retroperitoneal lymph node dissection; RT, radiotherapy; TNM, tumour–node–metastasis.

a Cancers in the Netherlands Cancer Registry are coded using the ICD-O valid at the time of diagnosis; first edition before 1993, second edition between 1993 and 2000 and third edition since 2001. Cancer types were categorized according to the histology-based AYA classification scheme developed by Barr et al. 2020. Malignancies were ICD-9 coded (ICD-code 186) between 1989 and 1995 and ICD-10 (ICD-codes C62) thereafter. Percentages may not total to 100% due to rounding.

b *P* values of differences between the diagnostic periods 1989-1999, 2000-2009 and 2010-2019. Pearson's X^2^ tests were used for categorical variables and Kruskal–Wallis tests for continuous variables.

c Tumour stage was classified as localized (T1-4, N0/Nx, M0/Mx or T0/Tx, N0, M0), regional lymph nodes (any T, N+, M0/Mx), distant metastases (any T, any N, M1) and stage unknown. Clinical-stage data were used to supplement missing/unknown pathological-stage data.

d Treatment proportions received at any time during the treatment process, irrespective of duration or completion.

e Additional ‘other’ treatment was received by *n* = 591 cases after orchiectomy and chemotherapy, which mostly included RPLND/metastasectomy in *n* = 418, chemotherapy in *n* = 119 and radiotherapy in *n* = 19 cases.

f Additional ‘other’ treatment was received by *n* = 9 cases after orchiectomy and radiotherapy, which mostly included chemotherapy in *n* = 6 cases.

g Additional ‘other’ treatment was received by *n* = 55 cases after orchiectomy and RPLND/metastasectomy, which mostly included chemotherapy in *n* = 50 cases.

h Additional ‘other’ treatment was received by *n* = 33 cases after orchiectomy and other treatment, which mostly included chemotherapy in *n* = 25 cases.

i Additional ‘other’ treatment was received by *n* = 65 cases after chemotherapy and orchiectomy, which mostly included RPLND/metastasectomy in *n* = 42 and chemotherapy in *n* = 17 cases.

j Additional ‘other’ treatment was received by *n* = 21 cases after chemotherapy and other treatment, which mostly included orchiectomy in *n* = 11 and chemotherapy in *n* = 8 cases.

k The total number of germ-cell tumours is the sum of the total number of seminomas and non-seminomas. The total number of testicular cancers is obtained by further adding the number of non-germ-cell tumours.

### Seminoma

#### Trends in incidence

**Figure 1:**
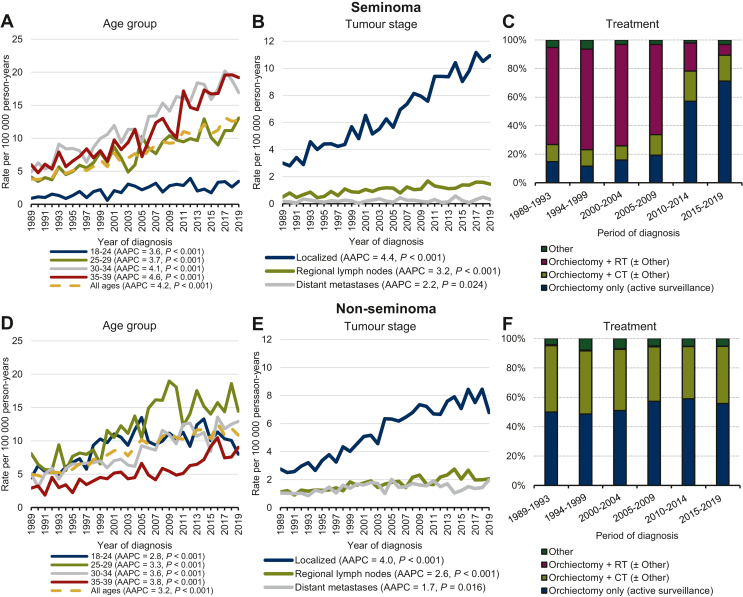
**Agegroup(A and D)and stage-specific(B and E)age-standardized incidence rates per 100 000 person-years, AAPC estimates and treatment(C and F)received by AYAs aged 18-39 years and diagnosed with testicular cancer in the Netherlands between 1989 and 2019.** Direct standardization of rates was done with weights from the 2013 European Standard Population. AAPC, average annual percentage change; AYAs, adolescents and young adults; CT, chemotherapy; RT, radiotherapy.

Seminoma incidence among AYAs increased annually on average with 4.2% and doubled from 4.9 in 1989-1999 to 11.4 per 100 000 person-years in 2010-2019. Significant increases in seminoma incidence were observed for all age groups, with the highest rates observed among those aged 30-34 years at diagnosis in 2010-2019. Increases in seminoma incidences were observed for all stages, but was most prominent for localized disease, increasing annually on average with 4.4% from 4.0 to 9.8 per 100 000 person-years from 1989-1999 to 2010-2019. The incidence of seminomas with regional lymph node involvement and distant metastases increased to 1.4 and 0.3 per 100 000 person-years, respectively, in 2010-2019 (Figure 1 and Table 2).

**Table 2:** Age-standardized incidence and mortality rates, and average annual percentage change (AAPC) estimates among adolescents and young adults (AYAs) diagnosed with testicular cancer at ages 18-39 years in the Netherlands between 1989 and 2019

Characteristics^a^	All testicular cancers				Seminomas				Non-seminomas			
	Age-standardized rates per 100 000 person-years^b^			AAPC (95% CI)^c^	Age-standardized rates per 100 000 person-years^b^			AAPC (95% CI)^c^	Age-standardized rates per 100 000 person-years^b^			AAPC (95% CI)^c^
**Period of diagnosis**	**1989-1999**	**2000-2009**	**2010-2019**		**1989-1999**	**2000-2009**	**2010-2019**		**1989-1999**	**2000-2009**	**2010-2019**	
**Incidence**												
Total	10.6	17.0	22.6	3.6∗ (3.2-4.1)	4.9	7.7	11.4	4.2∗ (3.8-4.5)	5.7	9.2	11.1	3.2∗ (2.6-3.7)
Age group (years)												
18-24	7.8	12.9	13.5	2.8∗ (1.8-3.8)	1.4	2.3	2.9	3.6∗ (2.4-4.8)	6.4	10.6	10.6	2.8∗ (1.6-4.0)
25-29	12.4	21.5	26.2	3.4∗ (2.5-4.4)	5.1	7.9	10.7	3.7∗ (3.0-4.4)	7.3	13.5	15.4	3.3∗ (1.9-4.6)
30-34	13.2	19.9	29.1	3.8∗ (3.1-4.5)	7.4	11.9	17.4	4.1∗ (3.5-4.6)	5.7	8.0	11.6	3.6∗ (3.0-4.3)
35-39	10.2	14.9	23.9	4.4∗ (4.0-4.8)	6.7	9.8	16.5	4.6∗ (4.0-5.2)	3.4	5.1	7.3	3.8∗ (3.0-4.6)
Tumour stage (TNM)^d^												
Localized	7.2	12.2	17.2	4.2∗ (3.7-4.7)	4.0	6.4	9.8	4.4∗ (4.0-4.9)	3.2	5.8	7.4	4.0∗ (3.3-4.7)
Regional lymph nodes	2.0	2.8	3.5	2.8∗ (2.4-3.2)	0.7	1.0	1.4	3.2∗ (2.4-4.0)	1.3	1.8	2.1	2.6∗ (2.0-3.3)
Distant metastases	1.4	1.8	1.8	1.7∗ (0.4-2.9)	0.2	0.2	0.3	2.2∗ (0.3-4.1)	1.2	1.6	1.5	1.7∗ (0.3-3.0)
Histological subtype												
Germ-cell tumours	10.6	16.9	22.5	3.6∗ (3.2-4.1)	NA	NA	NA	NA	NA	NA	NA	NA
Non-germ-cell tumours	0.0	0.1	0.1	NA	NA	NA	NA	NA	NA	NA	NA	NA
Carcinoma	0.0	0.0	0.0	NA	NA	NA	NA	NA	NA	NA	NA	NA
Sex cord	0.0	0.0	0.1	NA	NA	NA	NA	NA	NA	NA	NA	NA
**Mortality**												
Total	0.5	0.5	0.4	−1.5∗ (−2.6 to −0.4)	NA	NA	NA	NA	NA	NA	NA	NA
Age group (years)												
15-19	0.3	0.2	0.1	NA	NA	NA	NA	NA	NA	NA	NA	NA
20-24	0.6	0.5	0.3	NA	NA	NA	NA	NA	NA	NA	NA	NA
25-29	0.5	0.6	0.3	NA	NA	NA	NA	NA	NA	NA	NA	NA
30-34	0.5	0.6	0.6	NA	NA	NA	NA	NA	NA	NA	NA	NA
35-39	0.6	0.4	0.4	NA	NA	NA	NA	NA	NA	NA	NA	NA

CI, confidence interval; ICD-O, International Classification of Diseases for Oncology; NA, not applicable; TNM, tumour–node–metastasis.

a Cancers in the Netherlands Cancer Registry are coded using the ICD-O valid at the time of diagnosis; first edition before 1993, second edition between 1993 and 2000 and third edition since 2001. Cancer types were categorized according to the histology-based AYA classification scheme developed by Barr et al. 2020. Mortality data of reproductive organ cancers were retrieved from the cause-of-death statistics of Statistics Netherlands (CBS) for all individuals aged 15-39 years from 1989 to 2019. Malignancies were ICD-9 coded (ICD-code 186) between 1989 and 1995 and ICD-10 (ICD-codes C62) thereafter. Age group and stage-specific outcomes with insufficient data were omitted.

b Incidence and mortality rates were calculated per 100 000 person-years using the mid-year population size as person-time denominator and standardized with weights from the 2013 European Standard Population.

c AAPC and *P* value outcomes denoted with ‘NA’ could not be computed due to having zero counts in one or more individual years of diagnosis.

d Tumour stage was classified as localized (T1-4, N0/Nx, M0/Mx or T0/Tx, N0, M0), regional lymph nodes (any T, N+, M0/Mx), distant metastases (any T, any N, M1) and stage unknown. Clinical-stage data were used to supplement missing/unknown pathological-stage data.

∗ Indicates significant trends (*P* < 0.05).

#### Trends in treatment

**Figure 2:**
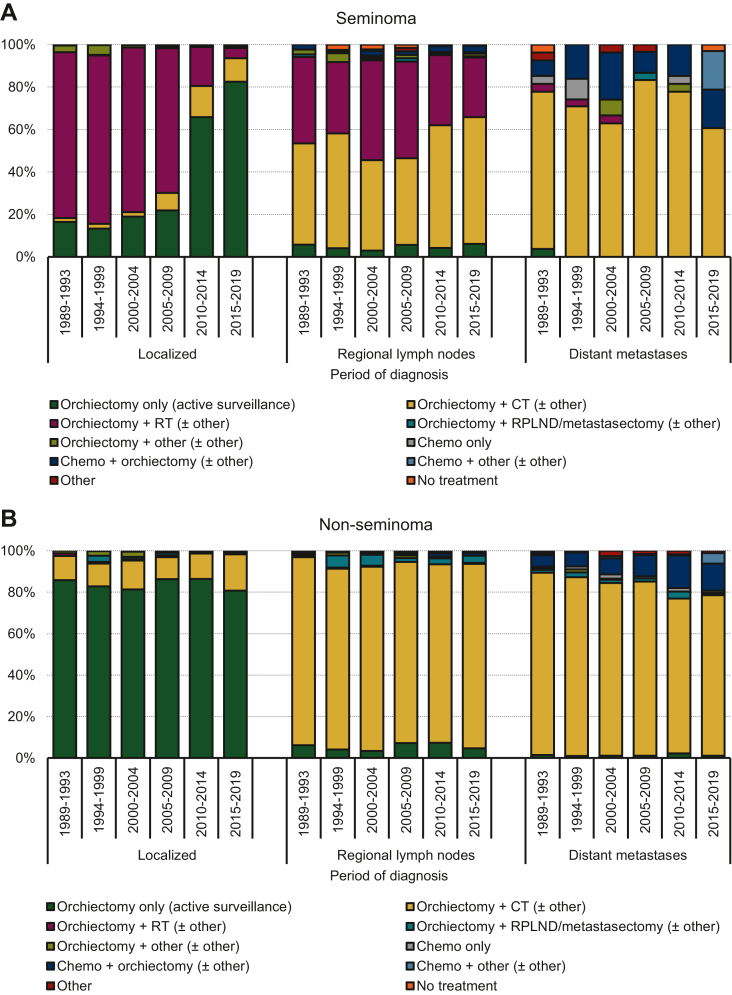
**Stage-specific treatment proportions received at any time during the treatment process, irrespective of duration or completion by adolescents and young adults (AYAs, aged 18-39 years) diagnosed with seminoma (A) and non-seminoma (B) testicular cancer in the Netherlands between 1989-2019.** AYAs, adolescents and young adults; CT, chemotherapy; RPLND, retroperitoneal lymph node dissection; RT, radiotherapy.

Treatment of AYAs with seminomas included orchiectomy as the initial treatment in most cases (≥97%). Chemotherapy was provided first in <3% of cases, whereas ≤0.2% did not receive any treatment (Figure 1 and Table 1). Treatment practices were similar across all age groups (Supplementary Figure S1, available at https://doi.org/10.1016/j.esmoop.2023.102231). AYAs (all ages) diagnosed with localized testicular seminomas received orchiectomy with adjuvant radiotherapy in around 70%-80% of cases until 2005-2009; afterwards its use declined to around 5% by 2015-2019. Meanwhile, there was a slight increase in adjuvant chemotherapy use from around 2% in 1989-1993 to 11% in 2015-2019. Use of orchiectomy with active surveillance increased with 66% since 1989-1993 and was received by 83% of AYAs diagnosed with a localized seminoma in 2015-2019. In AYAs with seminomas with regional lymph node involvement, radiotherapy use declined from 41% in 1989-1993 to around 28% in 2015-2019. Meanwhile, chemotherapy use increased by 12% up to 60% in 2015-2019. Seminomas with distant metastases were treated with chemotherapy in most cases, although there was a decline from 74% in 1989-1993 to 61% in 2015-2019. The remaining AYAs with metastatic seminomas received chemotherapy, followed by orchiectomy or other types of treatment (36% in 2015-2019). However, this only included 12 cases in total (Figure 2).

#### Trends in survival

The overall relative survival of AYA seminoma patients was >90% at any time, even at 20-year follow-up. Similar high outcomes were observed among all age groups. Survival by disease stage showed stable 5-, 10-, 15- and 20-year relative survival outcomes, with point-estimates >95% for AYAs that were diagnosed with seminomas that were either localized or had lymph node involvement in 2010-2019. Outcomes were lower, but point-estimates were still well above 85% in 2010-2019 for AYA seminoma survivors with distant metastases (Table 3 and Supplementary Figures S2 and S3, available at https://doi.org/10.1016/j.esmoop.2023.102231).

**Table 3:** Number at risk and relative survival outcomes with corresponding 95% CIs at 5, 10, 15 and 20 years of follow-up of adolescents and young adults (AYAs) diagnosed with testicular cancer at ages 18-39 years in the Netherlands between 1989 and 2019

Period of diagnosis^a^	*n* at risk^b^	1989-1999				*n* at risk^b^	2000-2009				*n* at risk^b^	2010-2019			
		RS (95% CI)^c^					RS (95% CI)^c^					RS (95% CI)^c^			
		5-year	10-year	15-year	20-year		5-year	10-year	15-year	20-year		5-year	10-year	15-year	20-year
All testicular cancers	3200	96.5 (95.7-97.1)	95.9 (95.1-96.6)	95.6 (94.6-96.4)	94.5 (93.5-95.4)	4164	97.9 (97.3-98.3)	97.5 (96.9-98.0)	97.1 (96.5-97.7)	96.0 (94.7-97.0)	5158	98.8 (98.4-99.1)	98.7 (98.0-99.2)	98.6 (98.0-99.1)	97.5 (96.6-98.2)
Age group (years)															
18-24	673	95.6 (93.7-96.9)	94.8 (92.7-96.3)	94.3 (92.2-96.0)	93.0 (90.6-94.9)	893	97.3 (96.0-98.2)	96.2 (94.7-97.4)	95.5 (93.8-96.8)	95.1 (92.9-96.8)	1010	98.3 (97.3-99.0)	98.4 (97.2-99.1)	98.2 (96.8-99.1)	97.5 (95.7-98.6)
25-29	890	95.7 (94.1-96.9)	95.1 (93.3-96.4)	94.4 (92.5-95.9)	93.6 (91.5-95.3)	1108	97.6 (96.5-98.4)	97.1 (95.8-98.0)	96.8 (95.4-97.9)	96.2 (94.2-97.7)	1400	98.9 (98.1-99.4)	98.5 (96.9-99.5)	98.0 (96.7-98.9)	97.1 (95.5-98.3)
30-34	949	97.6 (96.3-98.5)	96.8 (95.3-97.9)	96.9 (95.3-98.2)	95.5 (93.5-97.0)	1182	98.6 (97.7-99.2)	98.9 (97.9-99.5)	98.4 (97.2-99.3)	95.3 (92.0-97.6)	1502	98.7 (97.8-99.2)	98.4 (96.8-99.4)	98.7 (97.5-99.5)	96.7 (94.9-98.0)
35-39	688	96.8 (95.1-98.0)	96.9 (95.0-98.2)	96.4 (94.2-98.0)	95.8 (93.3-97.8)	981	97.7 (96.5-98.6)	97.5 (96.1-98.6)	97.4 (95.8-98.7)	97.3 (94.8-99.2)	1246	99.1 (98.2-99.6)	99.3 (98.0-100.1)	99.5 (98.1-100.4)	98.8 (96.8-100.3)
Tumour stage (TNM)^d^															
Localized	2163	99.2 (98.7-99.6)	99.0 (98.3-99.5)	99.0 (98.2-99.6)	98.4 (97.4-99.2)	3007	99.5 (99.1-99.7)	99.4 (99.0-99.8)	99.4 (98.7-99.8)	98.8 (97.5-99.8)	3917	99.9 (99.6-100.0)	99.9 (99.2-100.2)	99.7 (99.1-100.1)	99.2 (98.4-99.9)
Regional lymph nodes	588	96.3 (94.4-97.7)	95.2 (93.0-96.9)	94.1 (91.6-96.0)	92.3 (89.5-94.6)	691	97.4 (95.8-98.4)	96.9 (95.2-98.1)	96.7 (94.7-98.1)	94.9 (91.7-97.2)	807	98.9 (97.7-99.5)	99.3 (98.1-99.9)	98.4 (96.7-99.5)	96.2 (93.7-97.9)
Distant metastases	411	82.5 (78.4-85.9)	81.4 (77.2-84.9)	80.1 (75.7-83.8)	77.6 (73.0-81.6)	445	87.7 (84.2-90.4)	85.4 (81.7-88.5)	83.1 (79.0-86.5)	78.1 (71.3-83.6)	415	88.2 (84.6-91.1)	86.5 (81.1-90.4)	89.8 (85.8-92.8)	86.2 (81.7-89.8)
Histological subtype															
Germ-cell tumours	3191	96.5 (95.8-97.2)	96.0 (95.1-96.7)	95.6 (94.7-96.4)	94.5 (93.5-95.5)	4153	97.9 (97.3-98.3)	97.5 (96.9-98.0)	97.1 (96.4-97.7)	95.9 (94.7-97.0)	5140	98.8 (98.4-99.1)	98.7 (98.0-99.2)	98.6 (98.0-99.1)	97.5 (96.6-98.2)
Seminomas	1471	98.2 (97.3-98.9)	97.6 (96.5-98.4)	97.4 (96.2-98.4)	96.9 (95.5-98.0)	1915	99.3 (98.7-99.7)	99.4 (98.7-99.8)	99.1 (98.3-99.8)	98.3 (96.3-99.6)	2541	99.5 (99.0-99.8)	99.7 (99.1-100.1)	99.7 (98.9-100.2)	99.2 (98.1-100.0)
Age group (years)															
18-24	117	96.1 (90.3-98.5)	95.5 (89.5-98.3)	95.0 (88.7-98.1)	94.5 (88.0-97.9)	158	100.2 (100.2-100.2)	99.1 (95.3-100.1)	97.7 (92.8-99.6)	98.2 (93.2-100.1)	215	99.2 (96.0-99.9)	99.4 (96.3-100.2)	99.0 (94.0.-100.3)	99.5 (94.4-100.8)
25-29	365	97.3 (95.0-98.7)	96.9 (94.3-98.5)	96.5 (93.7-98.3)	96.6 (93.7-98.6)	407	99.0 (97.3-99.8)	98.8 (96.9-99.7)	98.5 (96.2-99.7)	98.2 (94.4-100.0)	569	99.3 (97.8-99.9)	99.6 (98.1-100.2)	98.9 (96.8-99.9)	98.9 (96.5-100.2)
30-34	536	99.1 (97.7-99.8)	98.0 (96.1-99.2)	98.2 (96.2-99.5)	96.9 (94.5-98.7)	703	99.5 (98.4-100.0)	99.9 (98.9-100.4)	100.0 (98.7-100.6)	97.0 (92.4-99.5)	898	99.5 (98.6-99.9)	99.9 (99.0-100.3)	100.5 (99.3-101.0)	98.8 (96.7-100.1)
35-39	453	98.4 (96.6-99.5)	98.2 (96.1-99.6)	97.7 (95.2-99.5)	97.6 (94.7-99.8)	647	99.1 (97.8-99.8)	99.1 (97.6-100.0)	98.9 (97.0-100.1)	100.2 (98.2-101.6)	859	99.6 (98.7-100.1)	99.8 (97.8-100.5)	99.5 (97.7-100.6)	99.7 (97.4-101.3)
Tumour stage (TNM)^d^															
Localized	1193	99.3 (98.5-99.8)	98.8 (97.8-99.5)	98.6 (97.4-99.5)	97.9 (96.4-99.1)	1594	99.6 (99.0-99.9)	99.8 (99.2-100.2)	99.9 (99.1-100.5)	98.8 (96.6-100.3)	2174	99.9 (99.5-100.1)	100.1 (99.4-100.5)	100.2 (99.4-100.7)	99.6 (98.4-100.5)
Regional lymph nodes	208	95.6 (91.6-97.8)	95.2 (91.0-97.7)	95.1 (90.5-97.8)	95.3 (90.4-98.3)	259	98.8 (96.3-99.8)	98.1 (95.1-99.5)	97.2 (93.5-99.2)	97.4 (92.9-99.9)	306	98.8 (96.1-99.8)	99.2 (96.6-100.2)	98.4 (95.0-100.0)	97.7 (93.5-100.0)
Distant metastases	58	84.8 (72.5-92.0)	81.7 (68.9-89.9)	82.4 (69.4-90.6)	83.3 (70.2-91.6)	57	93.4 (82.7-97.7)	92.0 (80.8-97.1)	87.8 (74.1-95.0)	88.8 (75.0-96.1)	60	87.4 (74.9-94.0)	87.8 (75.3-94.4)	92.4 (78.8-98.1)	93.5 (79.7-99.2)
Non-seminomas	1720	95.1 (93.9-96.1)	94.6 (93.3-95.7)	94.1 (92.7-95.2)	92.5 (91.0-93.9)	2238	96.6 (95.8-97.3)	96.0 (95.0-96.8)	95.5 (94.4-96.4)	94.0 (92.2-95.4)	2599	98.1 (97.5-98.6)	97.7 (96.5-98.5)	97.6 (96.7-98.4)	95.9 (94.6-97.0)
Age group (years)															
18-24	555	95.4 (93.3-97.0)	94.6 (92.3-96.3)	94.2 (91.7-96.0)	92.6 (89.9-94.8)	733	96.7 (95.0-97.8)	95.6 (93.8-97.0)	95.0 (93.0-96.5)	94.5 (91.9-96.4)	794	98.1 (96.8-98.9)	98.1 (96.7-99.0)	97.9 (96.3-99.0)	97.0 (94.9-98.3)
25-29	522	94.9 (92.6-96.6)	94.1 (91.6-96.0)	93.2 (90.5-95.3)	91.8 (88.8-94.1)	697	96.8 (95.1-97.9)	96.1 (94.2-97.4)	95.8 (93.8-97.3)	95.1 (92.3-97.1)	825	98.6 (97.4-99.3)	97.8 (95.1-99.2)	97.5 (95.6-98.7)	95.9 (93.5-97.6)
30-34	412	95.6 (93.0-97.3)	95.3 (92.6-97.2)	95.3 (92.4-97.4)	93.5 (90.2-96.1)	476	97.4 (95.4-98.6)	97.4 (95.3-98.7)	96.2 (93.6-97.9)	92.9 (86.9-96.6)	599	97.6 (95.9-98.7)	96.4 (92.4-98.5)	96.3 (93.7-98.0)	93.8 (90.5-96.3)
35-39	231	93.7 (89.5-96.3)	94.1 (89.8-96.9)	93.6 (88.9-96.8)	92.1 (86.8-96.0)	332	95.0 (92.0-97.0)	94.5 (91.1-96.7)	94.5 (90.9-97.1)	92.1 (85.9-96.2)	381	97.8 (95.4-99.0)	98.4 (96.0-99.7)	99.3 (96.4-100.7)	96.9 (92.4-99.7)
Tumour stage (TNM)^d^															
Localized	970	99.2 (98.2-99.7)	99.1 (98.1-99.8)	99.5 (98.3-100.2)	98.9 (97.5-100.0)	1412	99.3 (98.6-99.7)	99.0 (98.2-99.6)	98.7 (97.7-99.5)	98.8 (97.4-99.8)	1741	99.8 (99.3-100.1)	99.5 (98.1-100.2)	99.1 (98.2-99.8)	98.8 (97.5-99.7)
Regional lymph nodes	380	96.7 (94.2-98.2)	95.3 (92.4-97.2)	93.6 (90.4-96.0)	90.8 (87.0-93.7)	432	96.6 (94.3-98.0)	96.2 (93.7-97.8)	96.4 (93.9-98.1)	93.6 (88.9-96.7)	501	99.0 (97.4-99.7)	99.3 (97.7-100.0)	98.5 (96.3-99.7)	95.3 (92.0-97.5)
Distant metastases	353	82.1 (77.7-85.8)	81.4 (76.8-85.1)	79.7 (75.0-83.7)	76.7 (71.7-81.1)	388	86.8 (83.0-89.9)	84.5 (80.4-87.8)	82.4 (77.9-86.1)	76.7 (69.3-82.7)	355	88.4 (84.4-91.4)	86.3 (80.2-90.6)	89.4 (85.0-92.6)	85.1 (80.1-89.0)
Non-germ-cell tumours	9	75.4 (31.6-93.6)	75.9 (31.8-94.2)	76.5 (32.1-95.0)	77.6 (32.6-96.3)	11	100.3 (100.3-100.3)	100.8 (100.8-100.8)	101.3 (101.3-101.3)	102.2 (102.2-102.2)	18	92.9 (58.1-99.2)	NA	93.3 (56.1-100.2)	94.5 (56.8-101.5)
Carcinoma	2	50.2 (0.6-91.5)	50.6 (0.6-92.1)	51.1 (0.6-93.0)	51.8 (0.6-94.4)	4	100.3 (100.3-100.3)	100.7 (100.7-100.7)	101.2 (101.2-101.2)	NA	1	100.4 (100.4-100.4)	NA	101.4 (101.4-101.4)	102.9 (102.9-102.9)
Sex cord	7	83.8 (27.5-98.0)	84.3 (27.6-98.6)	85.0 (27.9-99.5)	86.2 (28.2-100.8)	7	100.3 (100.3-100.3)	100.8 (100.8-100.8)	101.4 (101.4-101.4)	102.2 (102.2-102.2)	17	92.3 (55.5-99.1)	NA	90.8 (46.1-99.9)	91.9 (46.7-101.1)

CI, confidence interval; ICD-O, International Classification of Diseases for Oncology; NA, not applicable; RS, relative survival; TNM, tumour–node–metastasis.

a Cancers in the Netherlands Cancer Registry are coded using the ICD-O valid at the time of diagnosis; first edition before 1993, second edition between 1993 and 2000 and third edition since 2001. Cancer types were categorized according to the histology-based AYA classification scheme developed by Barr et al. (2020). Malignancies were ICD-9 coded (ICD-code 186) between 1989 and 1995 and ICD-10 (ICD-codes C62) thereafter. Age group- and stage-specific outcomes with insufficient data were omitted.

b Number of cases alive at the start of follow-up when utilizing the cohort-approach.

c RS outcomes are denoted as ‘NA’ whenever they could not be calculated due to low case numbers. Period-approach was used to supplement the 15- and 20-year RS in 2010-2019. The cohort-approach was used otherwise.

d Tumour stage was classified as localized (T1-4, N0/Nx, M0/Mx or T0/Tx, N0, M0), regional lymph nodes (any T, N+, M0/Mx), distant metastases (any T, any N, M1) and stage unknown. Clinical-stage data were used to supplement missing/unknown pathological-stage data.

### Non-seminoma

#### Trends in incidence

Non-seminoma incidence annually increased with 3.2% from 5.7 in 1989-1999 to 11.1 per 100 000 person-years by 2010-2019. Significant increases in non-seminoma incidence were observed for all age groups. At 15.4 per 100 000 person-years, the highest non-seminoma incidence rates in 2010-2019 were observed among AYAs aged 25-29 years at diagnosis. Rising trends in overall non-seminoma incidence were observed for all disease stages. Highest rates and gains were observed for localized non-seminomas, increasing annually on average with 4.0% from 3.2 in 1989-1999 to 7.4 per 100 000 person-years in 2010-2019 (Figure 1 and Table 2).

#### Trends in treatment

Treatment of AYAs with non-seminomas remained largely unchanged and included orchiectomy as the initial treatment in most cases (≥97%). Chemotherapy was provided first in <3% of cases, whereas ≤0.2% did not receive any treatment (Figure 1 and Table 1). Treatment practices were similar across all age groups (Supplementary Figure S1, available at https://doi.org/10.1016/j.esmoop.2023.102231). AYAs diagnosed with localized non-seminomas received orchiectomy with active surveillance in 80%-86% of cases between 1989-1993 and 2015-2019, whereas adjuvant chemotherapy was provided in most cases otherwise. In non-seminoma cases with regional lymph node involvement, ∼90% received orchiectomy and chemotherapy regardless of the period of diagnosis. Non-seminomas with distant metastases were treated with chemotherapy in 88% of cases in 1989-1993, but this declined to 77% in 2015-2019. Meanwhile, use of chemotherapy followed by orchiectomy increased to 13% in 2015-2019 (Figure 2).

#### Trends in survival

There was a slight overall improvement in relative survival of non-seminomas since 1989-1999, with outcomes being well above 95% even 20 years after initial diagnosis in 2010-2019. Survival outcomes remained stable over time for most age groups, but some improvements were found among those aged 18-24 years when diagnosed with non-seminoma testicular cancer. Survival by disease stage showed mostly stable 5-, 10-, 15- and 20-year relative survival point-estimates that were >95% for AYAs that were diagnosed with non-seminoma testicular cancers that were either localized or had regional lymph node involvement in 2010-2019. For non-seminoma testicular cancer survivors with distant metastases, relative survival point-estimates remained between 85% and 90% up to 20-year follow-up in 2010-2019 (Table 3 and Supplementary Figures S2 and S3, available at https://doi.org/10.1016/j.esmoop.2023.102231).

### Trends in mortality

Testicular cancer mortality declined on average with 1.5% from 0.5 in 1989-1999 to 0.4 per 100 000 person-years in 2010-2019. In total, 374 male AYAs died due to testicular cancer in the Netherlands between 1989 and 2019 (Table 2).

## Discussion

This study showed a significant increase in germ-cell testicular cancers among AYAs in the Netherlands from 1989 to 2019 for both seminomas and non-seminomas. Radiotherapy usage for localized seminomas decreased considerably, whereas active surveillance and use of chemotherapy after orchiectomy increased. The relative survival of AYA testicular cancer patients did not improve in general, but was already high and remained well above 90% at 20 years of follow-up in most cases. Testicular cancer mortality among AYAs was low, but still declined since 1989.

Rising trends in testicular cancer incidence have been reported over the past decades among various industrialized countries by several studies (not AYA-specific) worldwide,^4^, ^5^, ^6^, ^7^, ^8^, ^9^, ^10^^,^^31^^,^^34^^,^^44^^,^^45^ for both seminomas and non-seminomas.^7^^,^^8^^,^^10^ In line with previous findings, germ-cell testicular cancers in this study comprised ∼95% of all testicular cancers among AYAs^46^^,^^47^ and were almost evenly split between seminomas and non-seminomas.^7^^,^^8^^,^^10^ For both subtypes, a rise in incidence was observed for all disease stages, with a majority of cases being localized. A shift in stage distribution towards more localized disease was already observed in the Netherlands among patients of all ages based on registry data between 1970 and 2009.^8^ Our data indicate that the shift towards localized testicular cancers has continued, as is supported by the proportional decline of seminomas and non-seminomas with regional lymph node involvement or distant metastases at diagnosis. Similar observations of increased localized testicular cancers were reported over a decade ago by other studies^48^^,^^49^ and likely results from improved early diagnosis following increased disease awareness among young men and general practitioners.^50^ Global differences in germ-cell testicular cancer incidence trends between ethnic groups have also been reported, but we were unable to investigate such trends due to unavailability of ethnicity data within the NCR.^9^^,^^31^

The outcomes of our treatment-specific analyses showed that AYA testicular cancer patients within the Netherlands have received treatment in close adherence to the clinical practice guidelines (e.g. NCCN and ESMO-EURACAN guidelines).^14^^,^^51^ Since 2005, use of adjuvant radiotherapy has drastically declined following the hallmark publication by Oliver et al.^52^ and subsequent guideline changes aimed at minimizing toxicity.^53^ Surveillance was now considered as standard and risk-adapted chemotherapy (one cycle of carboplatin) was discussed with patients. In line with these changes, curative radiotherapy in the Netherlands is nowadays only provided to AYA seminoma patients with limited regional lymph node involvement. Meanwhile, there was only a slight increase in adjuvant chemotherapy use, and orchiectomy followed by active surveillance has become the main therapeutic approach since 2010-2014, indicating that the omission of radiotherapy in testicular seminomas was not substituted by a different aggressive treatment regimen while survival rates remained high. Declined use of adjuvant treatment in clinical stage I seminomas is further supported by the findings of Boormans et al., who found that most patients did not relapse after orchiectomy alone regardless of rete testis invasion and primary tumour size, indicating that these risk factors with their low prognostic value for relapse should not drive the decision to provide adjuvant treatment.^14^ Still, risk factor-driven treatment decisions have become more common considering the observed rise in adjuvant chemotherapy use in seminoma patients with localized disease. Altogether, patients now likely suffer less from radiation-related adverse side-effects, meaning that the (long-term) quality of life of AYA testicular cancer patients has likely improved.

Comparable to previous studies,^2^^,^^8^^,^^54^, ^55^, ^56^, ^57^ testicular cancer survival outcomes in this study were typically high and leave little room for improvement, even at 20 years of follow-up. Five-year survival outcomes for metastatic disease in our study (>80% from 2000-2009 onwards) were more promising than in previous publications (e.g. non-seminoma: 70%^51^ and 78% for testicular cancers with distant metastases in the United States between 2002 and 2006^56^). The higher outcomes in more recent periods in our study could relate to increased guideline adherence and optimization of treatment, including centralization of care, in more recent time periods.^56^

It is well described in the literature that mortality rates due to testicular cancer at any age have been decreasing for decades and are now low (<1.0 per 100 000 person-years) in most countries.^8^^,^^9^^,^^31^^,^^34^^,^^44^^,^^58^ This is attributed to several chemotherapy-related discoveries, including the effectiveness of cisplatin in the 1960s and adoption of the combined bleomycin, etoposide and cisplatin (BEP) regimen in the 1980s.^8^^,^^11^ In the Netherlands, a sharp decline in testicular cancer mortality in men of all ages has been observed since the 1970s and 1980s, but rates were found to stabilize thereafter in some,^8^^,^^31^ but not all studies.^58^ We observed a steady decline in mortality rates despite low numbers between 1989 and 2019, amounting to an average of nine AYAs dying per year from testicular cancer in the Netherlands in 2010-2019. Previous studies have found that testicular cancer patients of all ages who were treated with platinum-based chemotherapy or radiotherapy between 1980 and 2009 had a significant higher risk of non-testicular cancer-related mortality compared to the general population.^15^ This was most prominent among testicular cancer patients who were diagnosed before the age of 20 years and the most important cause of death was second non-testicular cancer.^15^ Despite the current less-aggressive treatment regimens, the number of deaths following testicular cancer are most likely higher than the disease-specific mortality rates reported in our study and should not be underestimated.

The steep increase in testicular cancer incidence and their near-complete survival have resulted in a growing population of AYA cancer survivors who now face health issues throughout survivorship, including the development of subsequent malignancies,^59^, ^60^, ^61^, ^62^, ^63^, ^64^ cardiovascular diseases,^62^^,^^63^^,^^65^, ^66^, ^67^ symptomatic hypogonadism,^66^^,^^68^ sexual dysfunction,^66^^,^^69^ impaired fertility or fertility-related concerns.^69^ Multiple studies have shown an increased second cancer risk after chemotherapy and (sub-diaphragmatic) radiotherapy, with significantly lower long-term survival rates for those who developed a second malignancy (80% versus 40% at 30-year follow-up).^59^, ^60^, ^61^^,^^63^^,^^64^^,^^70^^,^^71^ Half of the identified second cancers included types that are more typically observed among middle-aged populations and it has been hypothesized that this might relate to treatment-induced premature ageing.^70^^,^^72^ The change in guidelines with a steep reduction in radiotherapy for seminomas is an important step forward in prevention of late effects. The same holds for the reduction in chemotherapy cycles for good risk metastatic non-seminomas and the omission or replacement of bleomycin in the BEP regimen by VIP (etoposide, ifosfamide, cisplatin) when indicated.^53^ Several studies also indicated increased gonadotoxicity risk after testicular cancer treatment with cisplatin- and carboplatin-containing chemotherapeutic regimens, resulting in impaired spermatogenesis.^73^^,^^74^ Moreover, reduced sperm count and pregnancy rates were observed after BEP regimen treatment, especially with increased number of cycles.^74^^,^^75^ Fertility-related concerns due to temporary or permanent reduction or loss of fertility are important long-term issues in cancer survivorship that require fertility-related counselling starting at the time of diagnosis.^73^^,^^76^ Altogether, these findings emphasize the need for evidence-based management strategies that optimize the follow-up care for testicular cancer survivors.^70^^,^^77^

This is the first AYA-focused study that provides a detailed assessment of testicular cancer by histological subtype, age, tumour stage and treatment based on three decades of high-quality data from the nationwide population-based NCR, which has near-complete coverage since 1989 and was systematically obtained by trained registrars, limiting possible selection bias. Despite the inclusion of various treatment combinations, older organ surgery codes within the NCR did not specify orchiectomy, but were registered as partial and total resection, or not further specified. The ‘orchiectomy’ groups may therefore contain other surgery types. Still, initial orchiectomy has been the mainstay testicular cancer treatment for decades and observed therapeutic trends were conform guideline expectations. A testicle is also a relatively small and clearly defined organ, limiting other surgical treatment methods and further supporting our approach. To best inform clinical practice, the period-approach was used to supplement long-term relative survival outcomes that could not be obtained through the standard cohort-approach for the 2010-2019 diagnostic period. Estimates obtained through the period-approach are typically higher, which may account for some observed increases in 15- and 20-year survival in the 2010-2019 period, whereas stable trends were found otherwise. Still, relative survival estimates obtained with the period-approach are more timely and representative of current-day patients.

In conclusion, the rising testicular cancer burden among AYAs in the Netherlands is dominated in equal parts by seminoma and non-seminoma germ-cell testicular cancers. There was a shift towards less-aggressive treatment regimens without negative survival effects. Meanwhile, testicular cancer mortality rates steadily declined over time. Evidence-based management strategies to improve patient-centred follow-up care for the ever-growing group of AYA testicular cancer survivors are needed.
