# Supplementary material for: The increasing burden of testicular seminomas and non-seminomas in adolescents and young adults (AYAs): incidence, treatment, disease-specific survival and mortality trends in the Netherlands between 1989 and 2019
Source: ESMO Open. 2024 Jan 19;9(2):102231. doi: 10.1016/j.esmoop.2023.102231 (PMC10937200; doi:10.1016/j.esmoop.2023.102231)
Supplement: Supplementary data [file mmc1.docx]

**Supplementary Materials:**

**The increasing burden of testicular seminomas and non-seminomas in adolescents and young adults (AYAs): Incidence, treatment, disease specific survival and mortality trends in the Netherlands between 1989-2019**

**Daniël J. van der Meer, Henrike E. Karim-Kos, Henk W. Elzevier, Marij Dinkelman-Smit, Jan Martijn Kerst, Vera Atema, Vicky Lehmann, Olga Husson and Winette T.A. van der Graaf**

**Supplementary Figure S1.** Age-specific treatment proportions received at any time during the treatment process, irrespective of duration or completion by adolescents and young adults (AYAs, aged 18-39 years) diagnosed with testicular cancer in the Netherlands between 1989-2019.

**Supplementary Figure S2.** Relative 5-, 10-, 15- and 20-year survival with 95% confidence intervals over time by age at diagnosis among adolescents and young adults (AYAs) diagnosed with seminoma and non-seminoma testicular cancer at age 18-39 years in the Netherlands between 1989-2019. The period approach was used to supplement the 15- and 20-year relative survival in 2010-2019. The cohort approach was used otherwise.

**Supplementary Figure S3.** Relative 5-, 10-, 15- and 20-year survival with 95% confidence intervals over time by stage at diagnosis among adolescents and young adults (AYAs) diagnosed with seminoma and non-seminoma testicular cancer at ages 18-39 years in the Netherlands between 1989-2019. Period approach was used to supplement the 15- and 20-year relative survival in 2010-2019. The cohort approach was used otherwise.

**Supplementary Figure S1.** Age-specific treatment proportions received at any time during the treatment process, irrespective of duration or completion by adolescents and young adults (AYAs, aged 18-39 years) diagnosed with testicular cancer in the Netherlands between 1989-2019.


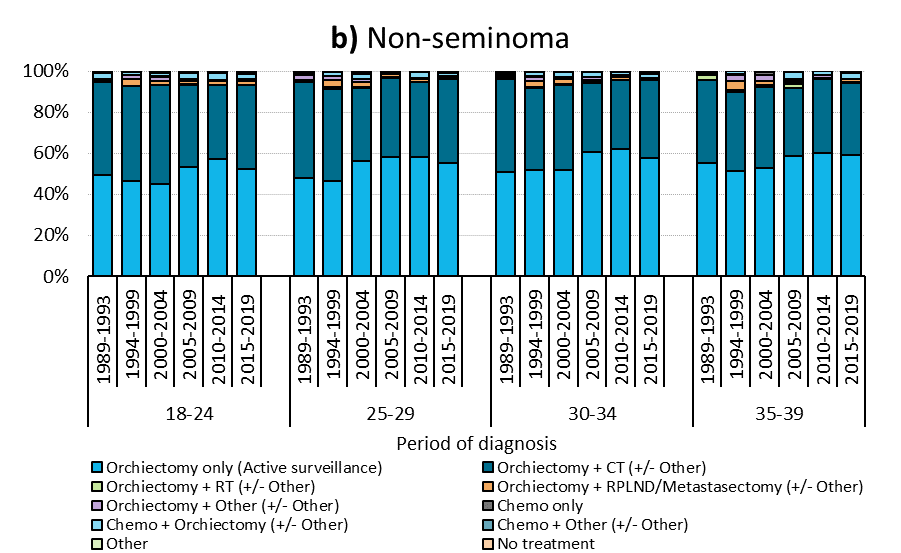

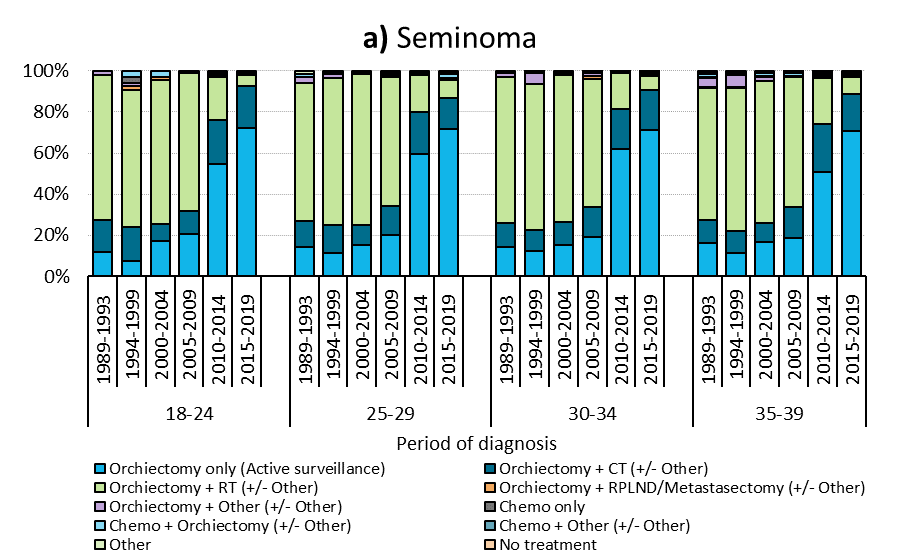

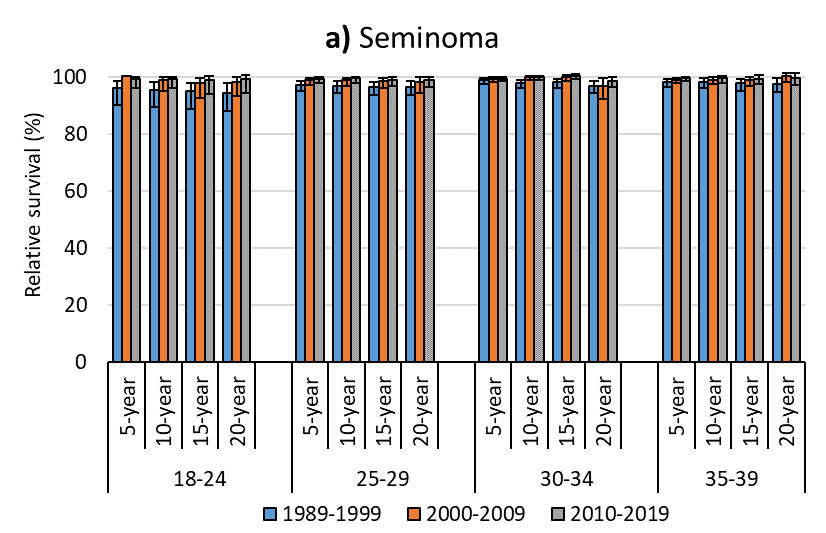

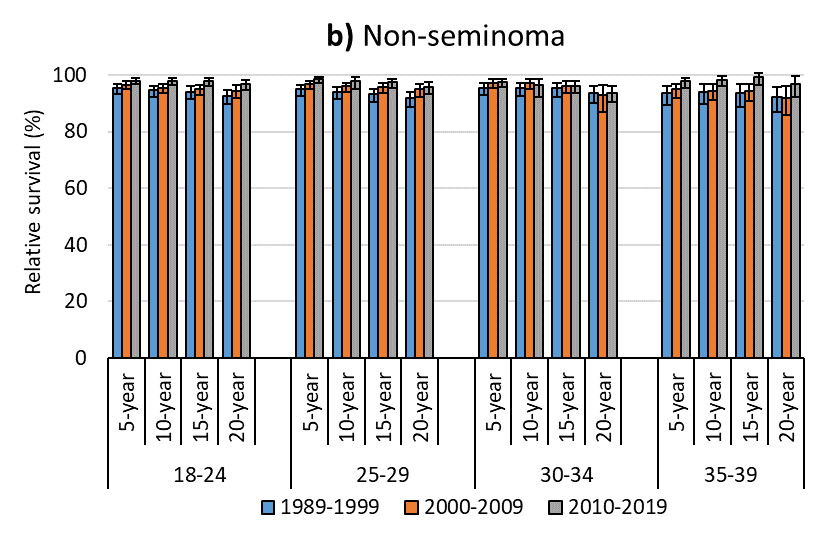


**Supplementary Figure S2.** Relative 5-, 10-, 15- and 20-year survival with 95% confidence intervals over time by age at diagnosis among adolescents and young adults (AYAs) diagnosed with seminoma and non-seminoma testicular cancer at age 18-39 years in the Netherlands between 1989-2019. The period approach was used to supplement the 15- and 20-year relative survival in 2010-2019. The cohort approach was used otherwise.


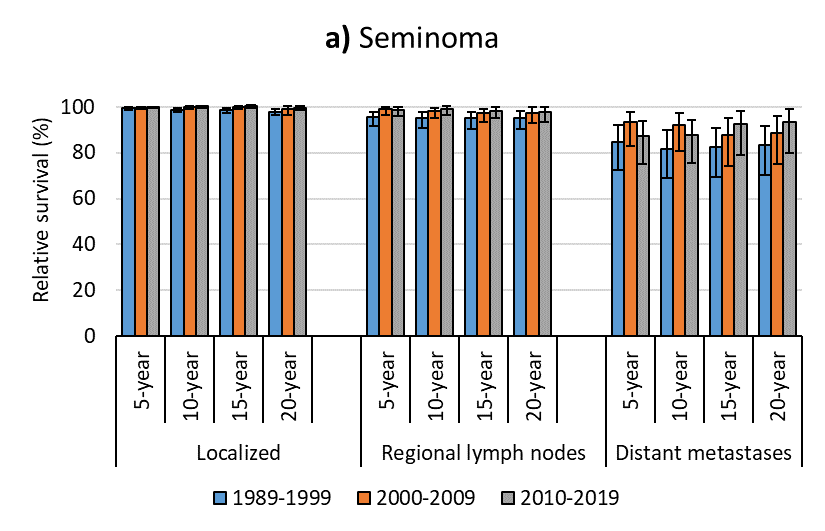

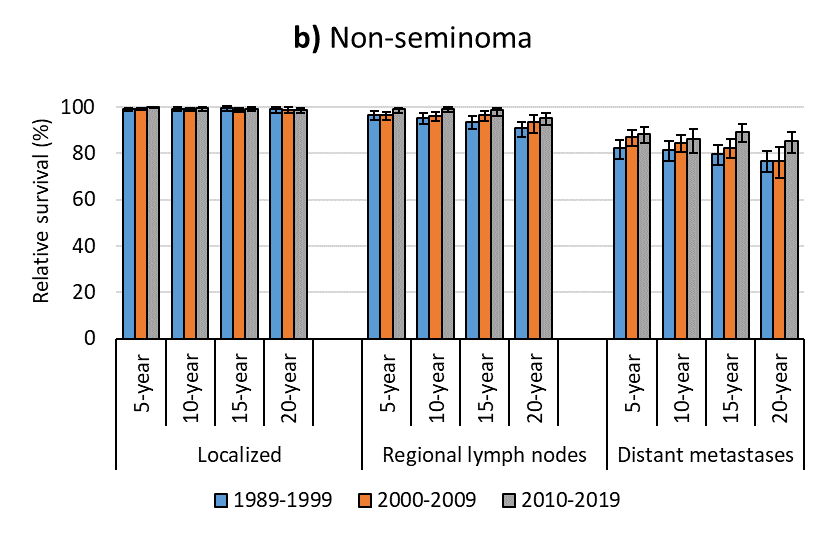


**Supplementary Figure S3.** Relative 5-, 10-, 15- and 20-year survival with 95% confidence intervals over time by stage at diagnosis among adolescents and young adults (AYAs) diagnosed with seminoma and non-seminoma testicular cancer at ages 18-39 years in the Netherlands between 1989-2019. Period approach was used to supplement the 15- and 20-year relative survival in 2010-2019. The cohort approach was used otherwise.
